# How do older patients and their GPs evaluate shared decision-making in healthcare?

**DOI:** 10.1186/1471-2318-8-9

**Published:** 2008-05-01

**Authors:** Danica Rotar-Pavlič, Igor Švab, Raymond Wetzels

**Affiliations:** 1 University of Ljubljana, Medical Faculty, Department of Family Medicine, Poljanski nasip 58, 1000 Ljubljana, Slovenia; 2 Centre for Quality of Care Research (WOK), Radboud University Nijmegen Medical Centre, Netherlands

## Abstract

**Background:**

Older persons represent a growing share of the population, yet very little is known about their specific healthcare needs, problems, and expectations. IMPROVE is an international research project that seeks to improve elderly persons' involvement in their healthcare. This paper analyzes perceptions of patient involvement by elderly patients and their GPs in family medicine in Slovenia.

**Methods:**

Semi-structured interviews with patients over 70 and their GPs were audio-taped and transcribed. The interviews were analyzed using qualitative content analysis.

**Results:**

Specific characteristics of old age must be taken into account in the involvement of older patients. It is important to know the patient's expectations and to communicate clearly with the patient. A trusting relationship between the GP and the patient is a prerequisite for involvement. GPs center involvement on the GP's side. Involvement of the elderly is linked to ethical dilemmas.

**Conclusion:**

Understanding the involvement of the elderly focuses more on building a relationship than on making decisions. It is reasonable to educate GPs and GPs' coworkers about caring relationships. Ethical aspects have often been treated in a theoretical manner, whereas empirical practice may be entirely different from theoretical premises. GPs and older patients must learn more about how to address their ethical dilemmas.

## Background

The traditional relationship and mode of communication between physician and patient have undergone radical changes over the last 40 years [1]. Better access to information about health, healthcare, and treatment options offer an opportunity for a new type of physician-patient partnership. As the user of health services, the patient has the primary role. The involvement and participation of patients in treatment decisions is increasingly important. Current models and measures of patient involvement in treatment decision-making tend to focus on communication within consultations and/or on the patient's use of information to consider the selection of one treatment option from a well-defined set [2]. Active participation leads to improved patient satisfaction, improved therapeutic compliance, better quality healthcare – including health status and satisfaction with care [3] – and a decrease in healthcare costs [4], partly due to reduced use of laboratory services and referrals [5]. Many factors may influence patients' preference for involvement [6], including some that relate to the patient, some to the physician, and some to the health system organization or cultural and historical background; all of these may change over time [7]. Sometimes attempts to achieve agreement between physicians and patients create conflict; for example, when patients' wishes are not in line with prevailing medical opinion. Patients may also interpret a physician's wish to involve them in decision-making as a sign of professional insecurity [8].

This article examines the needs and expectations of healthcare for the elderly. Life expectancy has increased. The proportion of the European population that is elderly (age 65 and older) increased from 13.9% in 1980 to 19.9% in 2000 (Eurostat population estimates). Older people's opinions about participation and involvement in medical treatment plans are poorly studied in Central and Eastern Europe. In Slovenia in 2003, 15% of the population was over 65, and by 2010 this share is expected to increase to 16.5% [9]. Following independence in 1991, there were several changes in the Slovenian healthcare system, including the introduction of a physician of choice that patients select as their family physician, changes in the health services financing system, and a different planning method [10]. Primary care in Slovenia is mainly provided by general practitioners (GPs), pediatricians, gynecologists, school physicians, and dentists [11], who control access to secondary care. Following healthcare reforms in Slovenia, the number of consultations with GPs increased considerably and the average duration of consultation decreased [12,13].

This paper is part of the international project IMPROVE, the purpose of which is to strengthen shared decision-making among patients over 70. Slovenia is also participating in this project. The first level of this three-year project consisted of analyzing the points of view and ideas about shared decision-making in healthcare among older patients and their GPs. The goal of this article is to analyze the role of shared decision-making in healthcare in Slovenia and thereby attain insight into the status of the active role of older patients in this Central European country.

## Methods

This study was conducted in Slovenia as part of the international project IMPROVE involving researchers from 11 countries: Austria, Belgium, Denmark, France, Germany, Israel, the Netherlands, Portugal, Slovenia, Switzerland, and the UK [14]. The project protocol defined uniform procedures in all participating countries, including a description of the sample of older patients and a description of the sample of GPs. As a research tool, we used a problem-oriented interview [15], structured with an emphasis on patient involvement and conducted in a manner that did not allow deviation to unrelated matters. During the interviews the interviewer was required to check that all questions were properly understood. The interview that we recorded with older patients started with the following introduction: ''As you have been informed by your GP, we and other GPs are interested in knowing whether patients wish to participate in decisions concerning their healthcare planning and treatment. What do you think about this?'' The follow-up questions are shown in Box 1[see Additional file 1].

The interview that we recorded with the GPs began with the following introduction: "In this project we wish to focus on how older patients participate in decision-making in general medicine. We would first of all like to determine which factors influence the use of methods to get older patients to participate in shared decision-making." The follow-up questions are shown in Box 2[see Additional file 2].

### Sampling and recruitment

The approximate number of participating older patients was defined at the international level. We interviewed 40 older patients stratified by age, sex, level of urbanization, and recent health status. The sample of older patients was chosen to achieve a balance by sex, age group (70 to 79 years old; 80 and older), place of residence (urban, suburban, rural), and health status (chronic disease, acute illness, life-threatening illness, and patients that had not consulted for a long time). Patients unable to speak Slovenian, in a terminal stage of a disease, or suffering from attention and concentration disorders were excluded. We had no difficulties recruiting older patients because their physicians of choice invited them to participate in the study. In Slovenia, the term *physician of choice *refers to a family physician that the patient has selected with his signature that ensures continuous and long-term care.

The sample of physicians that we interviewed in Slovenia was larger than in other countries. By including a larger number of physicians, we sought to include the points of view and evaluations of physicians that work in remote areas, those in private practice, those working in the public healthcare sector, school physicians, and those in partner or group practices. We included 26 physicians and recorded interviews with them.

Taped interviews were successfully conducted with 39 older patients. One patient interrupted the conversation during the interview, so his interview was excluded from the study. Most interviews with older patients were conducted in the patients' homes, and a minority at health centers. All of the interviews with GPs were recorded at their offices.

First of all, DRP listened to the tapes to check their quality. After this initial quality check, the tapes were transcribed by AA and ML, who had already participated in similar studies. DRP compared the transcriptions with the recordings. This was followed by a text analysis using the ATLAS computer program according to the following principles:

• Marking of relevant parts (statements, quotes) and encoding

• Linking of codes to relevant key content (categories, topics, subtopics)

• Repeated checking of quotes and codes (revision)

• Presentation of codes, quotes, and categories at a researchers' meeting

• Final creation of text files with final quotes, subtopics, and topics.

Special attention was paid to data validation: five primary texts of both patients and GPs were analyzed and coded by two independent researchers (DRP, MK). Good congruity between them was demonstrated. The international consistency of the coding was evaluated based on two translated interviews (one patient interview and one GP interview) that were sent to Richard Baker. No major international differences were found.

### Ethical approval

Before the study began, the entire international research protocol and the protocol translated into Slovenian were examined and approved by the National Medical Ethics Committee [16].

## Results

Thirty-nine older patients were interviewed between October 2000 and February 2001. Their ages ranged from 70 to 95. The interviews lasted 30 to 45 minutes. Interviews with 26 GPs were taped at their offices; these interviews lasted 20 to 40 minutes.

The recruited patients provided a detailed description of their perception of the relationship between GP and patient. They often interpreted involvement as a relationship in which the GP meets their expectations, whereas the GPs viewed these expectations as potential interference with their professional role. The patients evaluated involvement as a concept with four aspects:

• Mutual activity and improved relationship,

• Ethics,

• Relationship,

• Meeting expectations.

**Figure 1:**
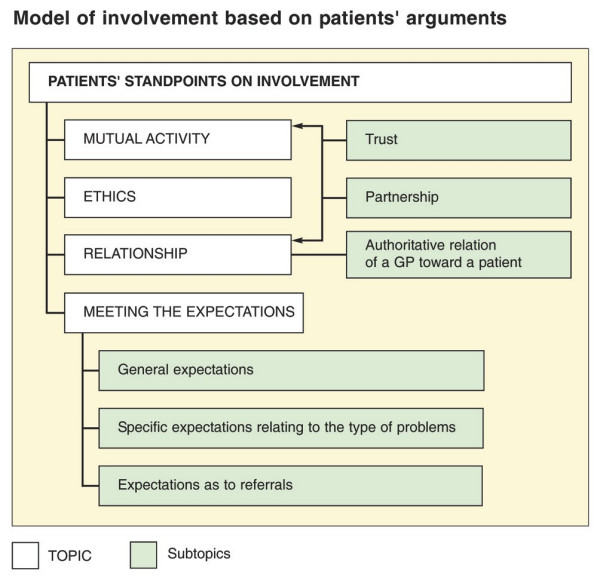
Model of involvement based on patients' arguments.

The synthesis of codes, categories, and themes depicted involvement as a concept, which is illustrated in Figure 1.

The majority of GPs felt that patient involvement in healthcare was beneficial and should be encouraged. An understanding of the meaning of involvement embraces several areas:

• Comprehension,

• Expert knowledge,

• Ethics,

• Communication,

• Relationship.

Most of the GPs defined involvement as a process of comprehension of dialogue during consultation. Involvement might be enhanced by the patient's relatives or the presence of a nurse. On the other hand, the presence of a third party was seen as a threat by some GPs. Patient's proposals in making healthcare decisions can interfere in their professional area.

"*Some elderly people actively interfere in matters, which means they're interfering not only with regard to themselves, but also in what the physician then says; then you need quite a lot of expert knowledge. They'd like to direct their treatment at a professional level.*" (GP, 202309)

"*We have problems with people, with patients, that think they know a lot about healthcare and want to decide for themselves and don't accept any explanation. Just because a friend told him 'no,' he believes his friend, even though he's not a GP with expert knowledge. I find it difficult to convince him otherwise.*" (GP, 204509)

**Figure 2:**
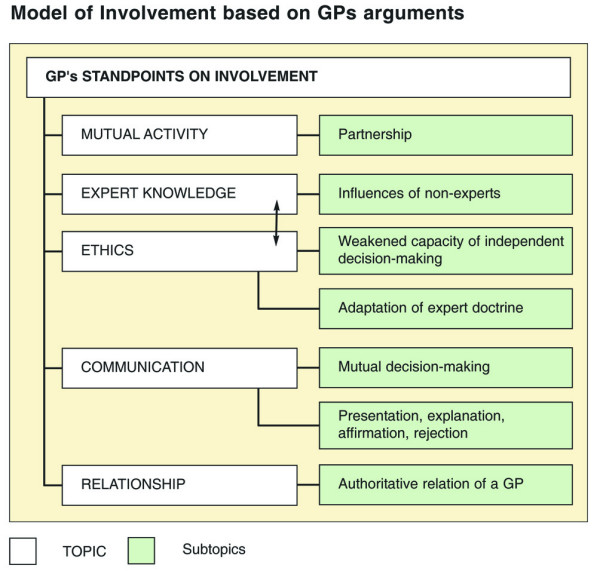
Model of involvement based on GPs' arguments.

The synthesis of codes, categories, and themes based on GPs' arguments is illustrated in Figure 2.

### 3.1. Involvement as mutual activity

The patients saw involvement as an improved relationship between the GP and patient that led to better treatment outcomes. Involvement in healthcare decisions is clearly dependent on the willingness and motivation to participate. Trust in the GP is essential. Even in the atmosphere of partnership, medical advice is not always accepted.

"*Oh, well, a patient gives his opinion. Absolutely. Why not? A patient knows himself. It's good for a patient to give his opinion about the treatment.*" (patient, 76 years old, 101309)

"*By all means a person has to have general contact with a GP. A patient talks to him. And there's something else: the GP says, 'I'm giving you this medicine, and I advise this, but if YOU don't do anything yourself, the treatment will be no good either.' So a person must do a lot himself and also trust the GP because nothing can be done without mutual consent.*" (patient, 71 years old, 102309)

The GPs consider involvement to be a process in which the GP advises the patient on diagnosis and treatment options. This process is based on consent. The following two quotes reflect examples of decision-making:

"*Especially when I familiarize a patient with various possibilities, options he has in diagnostics, treatment, and of course prognosis, and I obtain the patient's consent. Actually, I always inform him what it's all about, what disease he has or what disorder he's suffering from. I also explain the risk factors to him. I make suggestions and then ask him if he agrees. If he does, we cooperate; if he disagrees, I mark in his files that he doesn't agree.*" (GP, 201109)

"*By involvement I understand that the GP informs a patient about his diagnosis, about possible treatment, and about the prognosis, and that he offers him some options. For instance, that he'll undergo treatment with medicine or physiotherapy. In short, the GP has to talk to him about the treatment mode. A patient can also disagree with the suggested examinations and treatment, of course.*" (GP, 202209)

Comparatively few GPs questioned patients to find out what they had already done to treat their own problems, especially non-pharmacological measures.

"*By involvement I understand that a patient tells me what he'd do for himself. Yes, I always ask him what he's done, and then I ask him what he'd do, and then we make a common decision.*" (GP, 204309)

### Ethics

The elderly understood involvement to include respect for their reluctance to receive treatment at a clinic or hospital and their preference for the familiar domestic environment. They expect GPs to adapt their professional advice to their particular circumstances.

"*The GP examined me and he found out what was wrong. I gave blood, I went to have my urine checked. The GP gave me some medicine, but nothing improved. A few days later I came back for the same examinations, and he saw that the situation hadn't improved. He put his booklet on the table: 'You're going to the hospital.' Then I said: 'Doctor, I'm not going to the hospital. Just home, just home, I'm not going.' He wanted to know why. 'You know what, it's like this,' I said: 'I'm so old already.*"' (patient, 96 years old, 103409)

Several GPs treat elderly people in a stereotypical doctor-centered manner and do not allow them to become involved in healthcare. Others were concerned that patient involvement might lead them to deal with issues that were not relevant to optimum patient management.

"*The option that he (the patient) decides isn't good for him because he doesn't know himself.*" (GP, 203409)

### The physician-patient relationship

Some subjects felt that personal involvement in healthcare is not possible because the doctor is in charge and it is their job to follow instructions. Subordinate status in this authoritative relationship is shown by the following phrases used by patients: *to obey, permission to, to do what the GP says*. A similar interpretation of involvement was also found in the analysis of GPs' texts.

Interviewer: "*How much do you participate at the doctor's?*"

Patient: "*At the doctor's? Well, what the GP says, I should do, I do it. Let's take medicine, everything, I'd say I obey him.*" (patient, 81 years old, 107109)

"*The majority aren't prepared for this (involvement). The majority of elderly patients are used to practice in the past, when the GP decided on the therapy and the patient had no say about it. No one even thought about making their own decision, about expressing their points of view.*" (GP, 209109)

Trust in a GP is an important element of the relationship with a GP. The GPs did not highlight trust; they emphasized the tension and stress that patients experience when visiting a GP.

"*By involvement I understand that I trust a GP.*" (patient, 81 years old, 107109)

"*You have to trust a GP, and I think this is a good contact.*" (patient, 71 years old, 102309)

"*Patients are under some stress at the office. They're afraid and don't remember everything we say.*" (GP, 210509)

### Meeting patients' expectations and requirements, and expert knowledge

Some elderly patients evaluated involvement according to how well their expectations were met during the consultation.

Interviewer: "*Do you wish to be more involved in the decisions concerning your treatment?*"

Patient: "*So far, he *[the GP] *has done me a favor, he's really done me a favor.*" (patient, 76 years old, 112109)

"*By involvement I understand that I also tell the GP what I want, that I get it from him.*" (patient, 79 years old, 106209)

Many patients see referral to the patient's chosen specialist as an example of good physician-patient cooperation. Participation also means that a GP facilitates specialist examinations for patients wishing to be referred.

"*He *[the GP]* allows me everything, if I ask him for any kind of referral, anywhere. He's never refused to give one to me. I've always asked him for things that aren't stupid. He knew these doctors that I visited, and I had no problems with them because of that.*" (patient, 76 years old, 113109)

Some patients evaluated involvement passively, assuming the GP would address the patient's needs. From this perspective, the patient deliberately avoids taking any personal or even shared responsibility for his care.

Interviewer: "*Would you like to decide more and to participate in your treatment?*"

Patient: "*No, because I don't have many problems. I wouldn't want to make it more difficult, as far as the GP's work is concerned.*" (patient, 82 years old, 102109)

In patients' attempts to manifest specific needs and expectations, physicians see a danger that could impede healthcare. By expressing their nonprofessional expectations, a patient can "disturb" a professional treatment plan.

"*One disadvantage can be that a patient loses touch with reality. The GP is the one to tell him what reality is. The patient can go to extremes if he's overly involved. This means he deals with trivial issues that aren't vital, and trivial issues can become a major problem for a patient.*" (GP, 204309)

## Discussion

A clear understanding between the GP and the patient and a confident relationship between them are two important factors of healthcare [7,15,17]; this was confirmed in this study. Elderly patients stressed the importance of the GP-patient relationship, as has been previously reported [18]. The results of our study clearly express a need to build relations between doctors and patients; this can be explained by the fact that the structure of consultations has not significantly changed in the past decade, and that the building of partnership relations remains a minor part of the consultation [19]. Our study also expressed the importance of developing a relationship before taking action [20,21]. Older patients also wish to confidentially express trivial concerns and points of view that are important from a nonprofessional point of view, but are not part of narrowly-oriented professional medical treatment. Our research stands out because of its emphasis on an authoritative relationship. It is clear that older patients' healthcare is more oriented toward problem-solving phases of care; however, physicians feel uncomfortable if patients' subjective dimensions (i.e., points of view and expectations) are expressed in shared decision-making. This may be the result of insufficient instruction about patient participation, which is presented as a continuum between involvement and non-involvement at the undergraduate and graduate levels [22].

Our findings emphasize the diversity of perceptions and needs of the older population, and also confirm the model in which shared decision-making is defined as a continuum from the wish for the GP to make decisions for patients to active participation [22]. Patients differ in their needs for health-related information depending on their own priorities and their particular problems. Elderly people appreciate a relationship that embraces values such as trust, support, and discussion of feelings [23] and the individual as opposed to a disease orientation. Patients' desire for shared decision-making must therefore be defined individually [24]. The analysis of viewpoints showed mixed views on shared decision-making. Some patients want the GP to make decisions for them, whereas others would like to make decisions together, and still others stress one's personal responsibility for one's health. Shared decision-making is not possible (i.e., cannot be carried out) without co-determination by patients and GPs. It can occur only through the reciprocal relationships of dialogue and shared decision-making [25]. The communication method should be specifically adapted to the individual characteristics and preferences of the individual older patient.

Ethical issues were considered important by both older patients and GPs. Reliable information on the health issue and proper advice on treatment options is an ethical principle and a legal right [26,16]. One ethical principle that is of special importance in this regard is patient autonomy. It is a GP's professional responsibility to permit this, and it is influenced by political decisions and the characteristics of consumer society. An ethical dilemma is expressed not only with regard to patient autonomy, but also in adaptation and reduction of professionalism. "Forcing" GPs to change their professional doctrine and the perception of older patients as a special subgroup of people with limited cognitive abilities are current ethical issues. Tensions were discovered that arise due to GPs' focus on the best medical care for patients and expressions of patient autonomy. Studies [27] have shown that ethical aspects have often been treated in a theoretical manner, whereas empirical practice can be entirely different from theoretical points of departure.

Qualitative methodology as used here is particularly appropriate for research on perceptions, opinions, and personal experiences [17]. The "one interviewer – one patient" interviews were evaluated to provide added value from the words and statements that the interviewed subjects chose. The more delicate, personal, and emotional contents of the interview can be identified [17]. Direct observation of a group of older patients would not be possible without unanimous consent and would present unacceptable organizational difficulties. Nevertheless, the total sample met the stratification criteria and a broad range of views was achieved. Our findings cannot be generalized, but they do provide valuable indications and guides to further research.

## Conclusion

Understanding involvement of the elderly in healthcare is more focused on "building a relationship" than on making decisions. The perceptions and needs of elderly patients are characterized by considerable variation and diversity. Specific views and preferences regarding involvement in their healthcare should be explored during consultations. Dilemmas about patient autonomy and the adoption of a paternalistic approach come to the fore and there is a need to educate GPs and their coworkers on these issues. Physicians ought to provide elderly patients with the opportunity to express very personal points of view, expectations, and fears, even though (as sometimes trivial points of view and concerns) these do not have a significant influence on professional medical treatment of health problems.

In their continuing medical education, GPs should dedicate more time to the GP-patient relationship, patient participation, and patient responsibility for health.

The communication method should be adapted to the individual characteristics and preferences of the older patient. Reducing the GP's authoritarian attitude should not confuse people; patients should recognize this as an opportunity not only to express their expectations, but also to take greater personal responsibility for their health.

## Authors' contributions

All the authors participated in planning and designing the study. They drafted the manuscript and approved the final version. DRP and MW performed qualitative analyses. DRP wrote the manuscript.

## Pre-publication history

The pre-publication history for this paper can be accessed here:



## Supplementary Material

Additional file 1 Follow-up questions for the semi-structured patient interview. Questions are aiming to explore whether older patients wish to participate in decisions concerning their healthcare planning and treatment. Click here for file

Additional file 2 Follow-up questions for the semi-structured GP interview. Questions are aiming to explore which factors influence the use of methods to get older patients involved in shared decision-making. Click here for file
