# Supplementary material for: How do older patients and their GPs evaluate shared decision-making in healthcare?
Source: BMC Geriatr. 2008 May 1;8:9. doi: 10.1186/1471-2318-8-9 (PMC2386122; doi:10.1186/1471-2318-8-9)
Supplement: Additional file 1 — Follow-up questions for the semi-structured patient interview. Questions are aiming to explore whether older patients wish to participate in decisions concerning their healthcare planning and treatment. [file 1471-2318-8-9-S1.doc]

Box 1. Follow-up questions for the semi-structured patient interview

Follow-up questions:

- Have you ever had the feeling that you do not participate in your healthcare planning and treatment as much as you would like to?
- To what extent does your GP involve you in your healthcare planning and treatment, and give you the opportunity to be involved in the planning and decisions about your treatment?
- Can you describe an example of a situation or event?
- In your opinion, how could you be more involved?
- In general, would you like to participate more in your healthcare decisions?
