# Supplementary material for: How do older patients and their GPs evaluate shared decision-making in healthcare?
Source: BMC Geriatr. 2008 May 1;8:9. doi: 10.1186/1471-2318-8-9 (PMC2386122; doi:10.1186/1471-2318-8-9)
Supplement: Additional file 2 — Follow-up questions for the semi-structured GP interview. Questions are aiming to explore which factors influence the use of methods to get older patients involved in shared decision-making. [file 1471-2318-8-9-S2.doc]

Box 2. Follow-up questions for the semi-structured GP interview

Follow-up questions:

- How do you understand the idea of shared decision-making in healthcare?
- Can you give one or two examples?
- Thinking about your patients, what are the advantages for you of patients’ shared decision-making in healthcare?
- What obstacles prevent you from using methods to promote patient inclusion in healthcare at your office?
- What do you feel are the advantages and disadvantages of the tools that we use to promote shared decision-making?
- How do you view the future of patients’ shared decision-making in healthcare?
